# (Not) just policy success: Incorporating justice in policy evaluation

**DOI:** 10.1007/s11077-025-09588-3

**Published:** 2025-09-18

**Authors:** Nynke van Uffelen, Nihit Goyal, Amanda Martinez-Reyes

**Affiliations:** Delft University of Technology, Delft, Netherlands

**Keywords:** Distributive justice, Normative policy analysis, Policy evaluation, Policy success, Procedural justice, Recognition justice

## Abstract

Despite the recognition that policy evaluations are inherently normative as they are shaped by political and social values, justice is rarely addressed systematically in policy evaluation practice or research. By overlooking structural inequities and failing to scrutinize power dynamics, this omission risks hindering accountability, legitimizing injustice, and inhibiting policy learning. To help bridge this gap, we build on the policy success heuristic, which is a multidimensional approach for assessing programmatic, process, and political outcomes of public policy. Drawing on the philosophical literature on justice, we link three prominent categories—distributive, procedural, and recognition justice—with the dimensions of policy success. Based on this linkage, we propose a reflective framework that uniquely integrates justice principles into each dimension of the policy success heuristic. The framework can be applied ex-ante or ex-post to assess whether a policy is, or is likely to be, not only successful but also just, contributing to navigating the is/ought distinction at the heart of policy evaluation.

## Introduction

Public policies not only affect technical objectives but also fundamentally shape societal structures and influence justice, encompassing dimensions such as equity, fairness, and inclusion. Justice scholarship across research areas—including climate justice (Schlosberg & Collins, 2014), energy justice (Jenkins et al., 2016), environmental justice (Mohai et al., 2009), health justice (Ruger, 2012) and mobility justice (Mullen & Marsden, 2016)—demonstrates how policies can distribute benefits and burdens unevenly, exclude some stakeholders unfairly, or misrecognize marginalized groups. For instance, large-scale renewable energy projects like the Charanka Solar Park in Gujarat, India, illustrate how policies designed to address global challenges can perpetuate social inequities (Yenneti & Day, 2015; Yenneti et al., 2016). Similarly, climate adaptation policies in Nepal, while programmatically ambitious, have been critiqued for their technocratic framing, which excluded marginalized communities from decision-making processes, thereby undermining inclusion (Ojha et al., 2016). Such examples underscore the need for evaluations that explicitly address justice, ensuring that the pursuit of sustainability promotes inclusion and avoids perpetuating inequities.

The literature on public policy evaluation has been divided on questions of whether, how, and whose values should inform policy evaluation. At the heart of this debate is the conception of policy evaluation as a positivist versus a social constructivist exercise. Those with the former worldview typically advocate for ‘objective’ evaluations of goal attainment to foster policy learning and enhance policy effectiveness (Sanderson, 2002). In contrast, those in the latter camp view evaluations as inherently political (Taylor et al. 2005). Typically, they argue for more ‘subjective’ analyses that challenge policy objectives and incorporate inequities, power dynamics, and stakeholder perspectives in the process (House, 2019). In addition, they draw attention to the design and the conduct of the evaluation process itself and propose incorporating diverse values into the evaluation (House & Howe, 1999). Frameworks such as the policy success heuristic and realist evaluation aim to bridge this gap by recognizing both the positive as well as the normative aspects of policy evaluation (Marsh & McConnell, 2010; Pawson & Tilley, 1997).

Yet, even normative approaches to policy evaluation fail to systematically address justice. Although frameworks incorporating some lenses of justice—such as capability (Pereira et al., 2017), democracy, equity (Chapman et al., 2016), fairness (House, 1980), intersectionality (Heyen, 2023)—have been proposed, which one should be used in which context and to study which aspect of public policy remains unclear. Further, while existing frameworks highlight the distributional aspect and, to some extent, the procedural aspect of justice, they pay little attention to whether and how policies (mis-)recognize different societal groups. Third, in focusing primarily on the achievement (or not) of policy objectives (Gewirtz & Cribb, 2002; Leite et al., 2023; Silver, 2021), existing frameworks overlook structural inequities and fail to scrutinize power dynamics, hindering accountability and policy learning (Mohammed & Kuyini, 2021).

This study draws on political philosophy and applied justice research to address this gap. In general, justice is “the first virtue of social institutions” (Rawls, 1999).^1^ In this, institutions are understood in the broadest sense of the word, including “any structures or practices, the rules and norms that guide them, and the language and symbols that mediate social interactions within them” (Young, 1990). Applied justice research generally distinguishes among multiple categories of justice, including distributive, procedural, and recognition justice (Schlosberg, 2007). While existing research on policy evaluation has selectively borrowed concepts from this literature, our systematic engagement provides a clearer rationale for the selection of specific principles for policy evaluation, enables a more comprehensive assessment of policy justice, and helps identify the specific dimensions of public policies that enhance or worsen justice.

We propose a framework that integrates justice dimensions with the policy success heuristic (Marsh & McConnell, 2010). This heuristic is appropriate for our research objective as: i) it recognizes policy outcomes as multidimensional, encompassing not only the policy program but also the conduct of policy formulation and implementation and political control on the policy agenda; (ii) it is aligned with the inherently normative nature of evaluations as shaped by political and social values (Opfer, 2006); and (iii) it offers flexibility by allowing customisation to specific contexts, recognising that domain-specificities, local conditions, and historical inequities require tailored approaches (Siders, 2022). Yet, while the heuristic already incorporates aspects of justice, such as equity in programmatic evaluation and fairness in process evaluation (Compton et al., 2019; Compton &’t Hart, 2019), it does not systematically link policy success with justice, for example, the (political) repercussions of policies on marginalised groups. By incorporating this key aspect of justice, this study offers a practical tool for assessing whether policies are both successful and just. Additionally, it paves the way for a new research agenda on policy justice and contributes vocabulary to navigate the is/ought distinction at the heart of policy evaluation (Linquiti, 2024).

The article proceeds as follows. “On policy success” section reviews the state-of-the-art on the policy success heuristic, emphasizing its multidimensional approach while identifying its limited engagement with justice. “On justice in relation to policy” section conceptualises justice through three categories—distributive, procedural, and recognition—and positions them as key elements for evaluation. In the “Incorporating the notion of justice into policy success” section, we link these three types of justice with the three dimensions of policy success, proposing an integrated framework that bridges theoretical rigour and practical applicability. Finally, the “Discussion and conclusion” section discusses the implications of this framework for advancing both policy evaluation research and practice.

## On policy success

### The policy success heuristic: programmatic, process, and political outcomes

The policy success heuristic offers a multidimensional framework for evaluating public policies, encompassing programmatic, process, and political dimensions (McConnell, 2010). It emerged as a response to earlier approaches that equated policy success narrowly with goal attainment, overlooking broader implications. For instance, Bovens and ‘t Hart (1996) distinguished programmatic outcomes from political outcomes, highlighting that success in the former does not always translate to success in the latter (or vice versa). Marsh and McConnell (2010) further expanded this perspective by integrating process success, emphasizing that the way policies are developed and implemented influences their legitimacy and sustainability. The heuristic also recognizes that policy evaluation is both objective and normative, reflecting not only measurable outcomes but also the underlying political and social values that shape judgments of success (Bovens & ‘t Hart, 2016). Together, these dimensions provide a comprehensive lens for understanding policy outcomes beyond their immediate technical objectives.

The *programmatic* dimension refers to the operational aspect of policy and is largely evaluated based on the policy’s ability to achieve its intended societal outcomes. Programs represent the concrete forms of policy, combining tools of governance and resources to address specific problems (McConnell, 2010). A program is considered successful if its implementation aligns with stated objectives, desired outcomes are achieved, and it creates tangible benefits for the target group while meeting the specific criteria of its policy domain (McConnell, 2010). These criteria include technical feasibility, financial viability, and value acceptability (Kingdon, 1984), which may vary across domains—for example, economic efficiency in public budgeting versus secrecy in national security initiatives. Compton et al. (2019) emphasize additional criteria for programmatic success, such as a well-developed and empirically feasible public value proposition, a sound theory of change, and the equitable distribution of costs and benefits. Programmatic success also depends on selecting the ‘right’ policy target (Green et al., 2014), aligning policy design with target behaviour (Howlett, 2018; Weaver, 2014, 2015), and minimizing anomalies through monitoring and policy learning (Hall, 1993).

The *process* dimension pertains to how societies define problems, specify alternatives, adopt decisions, implement programs, and evaluate outcomes (McConnell, 2010). This dimension encompasses the broader design, decision-making, and delivery processes that underpin collective policymaking. It considers key questions about whom to consult, why, how, and when during the policy process. Success in this dimension is determined by criteria such as preserving policy goals and instruments, conferring legitimacy on the process, building a sustainable coalition, and limiting opposition to the process (McConnell, 2010). Compton et al. (2019) emphasize rigorous deliberation and the balanced consideration of diverse evidence, expertise, and advice as additional hallmarks of process success. Factors such as the degree of centralization (Durant & Diehl, 1989), how policy alternatives influence interests (Beland & Cox, 2016), social appropriateness (Green, 1997), and consistent political communication (Zahariadis & Exadaktylos, 2016) play a crucial role in shaping process outcomes.

The *political* dimension acknowledges that policies have political repercussions (McConnell, 2010). This dimension explicitly posits that even policies with limited programmatic or process achievements can yield significant political dividends if they enhance public perception or consolidate power (Bovens et al., 2001; Marsh & McConnell, 2010). Political success is assessed based on criteria such as the government’s ability to control the policy agenda, the sustenance of its political and administrative values, and the level of opposition to the political benefits of the policy (McConnell, 2010). Additionally, Compton et al. (2019) emphasize the enhancement of political capital for policymakers and the improved organizational reputation of public agencies. The likelihood of political success depends on factors such as the policy’s salience, its alignment with public mood, the government’s ability to claim credit or avoid blame, and the balance of interests among stakeholders (Dunleavy, 1986; Howlett, 2012; May, 1992; Weaver, 1986). Effective agenda control, achieved by neutralizing competing political windows and limiting access to alternative institutional venues (Baumgartner & Jones, 1993; Kingdon, 1984), can further strengthen the political standing of a policy (Goyal, 2021).

The policy success heuristic represents a significant advancement in policy evaluation. Its integration of programmatic, process, and political dimensions enables holistic assessments, moving beyond traditional approaches that focus solely on the policy program. A key strength of the heuristic lies in its acknowledgment of the dual nature of evaluation, blending ‘objective’ outcomes with normative judgments, for example, regarding equity, fairness, and legitimacy. By framing success as a spectrum rather than a binary outcome, the heuristic accommodates nuanced evaluations that recognize partial successes or areas for improvement. Additionally, the heuristic’s adaptability allows it to accommodate diverse policy domains and governance contexts, offering a versatile tool for analysing policies with varying criteria for success. This flexibility ensures its relevance to domain-specific challenges, local contexts, and evolving societal expectations. Furthermore, its acknowledgment of the temporal nature of success recognizes that performance along programmatic, process, and political dimensions can evolve over time in response to contextual factors and political dynamics (Goyal, 2021; Newman & Head, 2015). However, despite these strengths, its engagement with justice remains implicit and uneven, as explored in the following section.

### On the relationship between policy success and justice

Despite its multidimensional approach, the policy success heuristic does not systematically address concerns regarding justice. For decades, critiques of policy evaluation have emphasized the importance of the normative dimension in policy evaluation. Kerr (1976), for example, highlighted that policies can fail morally even when they are operationally successful. More recently, Reslow (2017) underscored the need to incorporate normative justification as a dimension in policy evaluation. These critiques point to the broader need to evaluate not only whether policies achieve their goals but also whether they do so in a way that is equitable, fair, and inclusive. While opposition to policy has been considered a criterion for policy success (McConnell, 2010), just policies can be met with opposition, while unjust policies sometimes do not result in opposition (Taebi, 2017). Further, the *experience* of the absence of injustice cannot be equated with the *actual* absence of injustice (ten Caat et al., 2024; van Uffelen & ten Caat, 2025). Also, as McConnell et al. (2020) observe, the question of “success for whom” remains underexplored, leaving a significant gap in understanding how policies distribute benefits, include marginalized voices and shape political agency.

The *programmatic* dimension focuses on achieving intended outcomes and delivering benefits to target populations. However, it often neglects inequalities in who benefits and who bears the costs (Newman, 2014; Newman & Head, 2015). While Compton and ‘t Hart (2019) take a step forward by incorporating equity into programmatic evaluation, their approach remains incomplete. For example, Jancovich and Stevenson (2021) argue that fairness extends beyond the equitable allocation of resources to include whether policies recognize and respect the lived experiences of affected groups. Programmatic evaluations that focus solely on measurable outcomes risk alienating marginalized communities by neglecting the subjective realities of those impacted. To address these limitations, evaluations must move beyond technical criteria to consider whether policies empower or exclude those they aim to benefit.

The *process* dimension evaluates how policies are developed, deliberated, and implemented, emphasizing legitimacy, representativeness, and procedural integrity. While McConnell (2010) identifies legitimacy as a key criterion, his heuristic does not explicitly address whose voices are included or excluded in the decision-making process. Compton and ‘t Hart (2019) emphasize deliberative rigor and evidence-based decision-making as hallmarks of successful processes. However, these criteria are grounded in liberal democratic traditions, which may not translate to governance contexts with differing norms of representation or decision-making. For example, some societies may hold that decisions should be made by the wisest members of a society or dictated by religious leaders. Moreover, existing frameworks often overlook critical questions about how power dynamics shape who participates, who is excluded, and whose interests are prioritized. Addressing these gaps requires a more nuanced approach to evaluating policymaking processes, one that accounts for varying governance contexts and interrogates how inclusivity and fairness are operationalized in practice.

The *political* dimension evaluates policies based on their impact on political capital and agenda control (McConnell, 2010). However, its focus has traditionally been limited to the interests of governments and policymakers. As McConnell et al. (2020) note, policies can have significant political repercussions. For example, Québec’s preferential language laws, while politically advantageous for the majority French-speaking population, alienated English-speaking minorities, leading to economic disenfranchisement and widespread emigration (Endleman, 2009). This case illustrates how policies that appear politically successful for governments can simultaneously disempower marginalized communities. Mohammed and Kuyini (2021) argue that political evaluations must interrogate power dynamics to assess whether policies reinforce or challenge systemic inequities. Broadening the political dimension to account for societal impacts would help address questions of fairness and inclusion.

Together, these critiques underscore the need for a systematic framework that explicitly addresses questions of fairness, inclusivity, and empowerment across the policy success heuristic’s three dimensions. Incorporating justice into policy evaluation requires expanding and diversifying evaluative criteria. In the next section, we introduce a conceptualization of justice to take a step towards addressing this gap more systematically.

## On justice in relation to policy

‘Justice’ is a fundamentally plural and contested^2^ concept. Several scholarships, such as environmental justice, spatial justice, and energy justice, distinguish between different *categories* of justice, including distributive, procedural, and recognition justice (Heffron & McCauley, 2018; McCauley et al., 2013; Schlosberg, 2004, 2007). These three categories of justice are a useful heuristic, as they break down the complex concept of ‘justice’ into three parts that pertain to different facets of policies, namely the distributive outcomes; the decision-making procedures; and the institutionalized values and normative assumptions. Before incorporating these categories of justice into the policy success heuristic, we briefly outline for each category of justice: (i) its meaning; (ii) plurality and contestation within that category; and (iii) illustration of its application to public policy.

### Distributive justice

Distributive justice is about the just distribution of goods and services, and of burdens and benefits. This category of justice is sensitive to who receives the burdens and benefits of policies. In general, distributive injustices imply that certain social groups (structurally) receive too few benefits while bearing too many burdens in comparison to others. Thus, almost all matters of taxation, public spending, and regulations are paradigmatic of distributive justice issues.

Within political philosophy, there are many different conceptions or *principles* of distributive justice, representing different standards by which policies can be assessed. One view is that if the benefits of the policy are acquired legitimately, then distributive inequalities resulting from the policy are just (Nozick, 1974). However, such a view is heavily critiqued, and most philosophers consider the end-state for evaluating distributive outcomes. The principle of *strict equality*, for example, argues that everyone should receive the same amount of goods, services, or benefits and burdens (Walzer, 1983). While most scholars agree that some degree of inequality in a society is justified, exactly how much and which inequalities are justifiable is a matter of much debate (Miller, 2017). Principles such as *equality of outcomes* or *equality of opportunities*—which contrast *equality* and *equity*—have, therefore, been proposed. John Rawls (1999), for example, suggested with his *difference principle* that inequalities are justified if they benefit the least well-off. Consequentialist theories, such as utilitarianism, for example, evaluate the outcomes or consequences of distributions in terms of well-being or pleasure. Other theories of justice focus on *needs or dignity*, and as such, they suggest minimal criteria for just distributions. Illustratively, the capability approach outlines several capabilities that all humans should have, and if this is not the case, then society ought to redistribute its goods (Nussbaum, 2011; Sen, 2009).

Different principles offer competing criteria for distributive (in)justice, potentially yielding divergent normative evaluations of the same policy. For example, Wood and Roelich (2020) show that different principles, namely utilitarianism and the capability approach, applied to the case of a hydropower project in India, lead to different evaluations and policy recommendations. Following utilitarianism, the project can be justified, as it produces significant benefits in the form of renewable energy and wealth, which weighs against the disadvantage of the massive relocation of vulnerable groups in the area. However, following the capability approach, the project would not be justified, as it harms several capabilities of vulnerable people in the vicinity. A careful consideration of the appropriate principle of distributive justice in a specific context is, therefore, necessary.

### Procedural justice

Procedural justice is about the design and execution of the decision-making process, i.e., who makes the decisions, who participates, who sets the agenda, and who is excluded from this process. There are various approaches to decision-making participation, which can be perceived as just. Yet, in practice, the appropriate form of participation is defined according to the objective of participation, including giving information, consultation, deliberation, or empowerment (Davidson, 1998).

In political philosophy, key conceptions of procedural justice are often related to democratic principles (see the philosophy of Immanuel Kant) and the separation between legislation, administration, and jurisdiction (see, for example, Montesquieu’s *trias politica*). While the *one-person-one-vote-principle* might seem straightforwardly just, there is much discussion about who should get a vote. For instance, decisions made in countries often affect people and environments beyond the national borders. To incorporate this concern, the *all-affected principle* is proposed, which evaluates a decision-making process as just if all-affected actors have been given a voice (Warren, 2024).

Further, most countries in the world are *indirect or representative democracies*, which are generally based on the belief that procedures are considered just if they are made by democratically chosen representatives (Arnstein, 1969). Next to these procedural conceptions of democratic principles, s*ubstantive democracy notions* pose an additional criterion to what is considered democratic, for example, the outcomes of procedures need to adhere to basic rights and liberties (Tamanaha, 2004). Even so, to avoid a tyranny of the majority and to protect minorities, decision-making forums may be supplemented, embodying principles such as *positive discrimination* (Young, 1990). Moreover, philosophers have posited many principles for just procedures, such as the *veil of ignorance* (Rawls, 1999), or *guidelines for ideal deliberation* (Habermas, 1990).

Besides democratic principles of procedural justice, there are also radically different conceptions that may be appropriate in specific contexts. For example, the more technocratic governance of certain technologies, such as nuclear waste disposal or climate engineering, is often justified on the grounds that some decisions are best made by experts (Hannis & Rawles, 2013; Winner, 2017). Moreover, some decision-making procedures in non-Western contexts seek to incorporate local traditional principles that would otherwise be overlooked by dominant Western values. For example, an assessment of geothermal energy projects in Indigenous territory in New Zealand combined Māori’s spiritual, customary, beneficial, and political principles with Western criteria such as economic, social, and environmental (Hikuroa et al., 2010). Consequently, procedural justice should not be reduced to stakeholder inclusion without taking the context into account.

### Recognition justice

Recognition justice has its origins in a resistance against the ‘distributive paradigm’ in political philosophy, in which social justice is reduced to the distribution of goods and services (Young, 1990). The argument for recognition is that justice should also include concerns for unjust cultural norms and values that are institutionalized, such as racist, sexist, ableist, ageist, or colonial norms. Such norms can also be unjust, and they can underlie unjust distributions, yet they are not usually considered through a distributive justice lens.^3^ In other words, some injustices have roots in unjust social norms and relations that are institutionalised, and these injustices cannot be reduced to maldistributions or exclusions in decision-making, thus leading to the distinct category of recognition justice. Therefore, institutions should also ‘recognise’ people, and if they fail to do so, it leads to injustice.

In general, people need, want, or deserve to be recognized in three modes (Honneth, 1995; van Uffelen, 2022; van Uffelen & Santos Ayllón, 2025): (i) *love*, as we all want to be loved by a small circle of friends, family, and life partners with whom we share deep and meaningful relationships, and we want our bodily integrity to be unharmed (micro-level); (ii) *status order,* as we want to be valued for our group identities and contributions to society (meso-level), and; (iii) *dignity,* that is, to be respected through laws in the capacity of being human with autonomy and intrinsic dignity (macro-level). These three modes translate into three different ways of misrecognition, in other words, misrecognition can pertain to policies that hinder interpersonal relationships and bodily integrity; implicitly devalue specific identities, skills, or professions; and fail to respect human dignity and autonomy. For example, a policy that dictates that gay marriage is forbidden, implicitly labels gay people as inferior or faulty, which is misrecognition (interfering with relations of ‘love’); a policy that is racist (or implicitly contributes to racism) is misrecognition through status order; and a government that dismisses human rights institutionalizes misrecognition through dignity.

Two distinct principles of recognition justice have been proposed in the literature. In other words, policies can be evaluated as unjust because they misrecognize people by two different yardsticks (van Uffelen, 2022). The first is an *undistorted relation-to-self* (Honneth, 1995)*.* Honneth assumes that people’s identities and their autonomy are relationally constituted, meaning that being (mis)recognized by other people co-shapes how we see ourselves. Through recognition of love, we gain self-confidence; recognition through status order grants us self-esteem, and recognition through dignity gives us self-respect. Thus, if policies embody (structural) misrecognition that contributes to an *undistorted relation-to-self*, then they are unjust. The second is the principle of *participatory parity in social life*. Avoiding reliance on psychological experiences for assessments of injustice (Fraser & Honneth, 2003), Fraser (2000) argues that if institutions hinder people’s ability to be peers in social life, then the institutions are unjust. Note that participatory parity in social life goes beyond mere participation in decision-making procedures; it plays out in all aspects of social life.

In a sense, the recognition justice tenet represents a different ‘lens’ to look at institutions, as it considers not only the decision-making procedures and the outcomes, but the social norms and relations embedded in the policy outcomes, processes, and the broader environment. As a result, recognition justice is deeply intertwined with, and often underlies, distributive and procedural justice. For example, Bailey et al. (2017) argued that health inequities cannot fully be explained by social and economic factors and that structural racism plays a significant role in the constitution of this injustice. Similarly, Terry (2009) contended that climate justice cannot be achieved without paying attention to institutionalized gender relations and conceptions. Assessing public policies from a recognition lens is, therefore, critical for uncovering hidden injustices.

## Incorporating the notion of justice into policy success

Policies can be successful yet unjust, or vice versa–in other words, policy success does not guarantee that policies are just, and just policies do not guarantee policy success. Consequently, a holistic policy evaluation ought to focus on both policy success and justice, and ‘just policy success’ occurs only when policies are successful *and* just.

When linking policy success and justice, three different dimensions of policies can be assessed as (un)just, namely: (1) the *policy program:* are the policy objectives, instruments, and their outcomes just?; (2) the *policy process:* are decisions being made, implemented, and evaluated in a just way?; (3) the *political outcomes:* are the outcomes in terms of the political power of actors and their standing in society just? In this section, we link each dimension of policy success to justice, with the aim of identifying the relevant categories of justice when assessing ‘just policy success’ for each element. Based on this linkage, we identify a set of guiding questions that can support analysts, policymakers, evaluators, and stakeholders in navigating the key aspects of justice-oriented evaluation.

### Justice-incorporating notion of program success

To recall, program success occurs if the policy attains its preset goals and outcomes. Some of the established criteria for programmatic success related to goal attainment include realizing intended consequences and minimizing opposition to the program (McConnell, 2010). In a justice-incorporating notion of program success, however, program success occurs when policies attain their goals, *and if those goals are just*. In this light, some of the established criteria must be amended to incorporate justice. For example, McConnell’s criterion of “creating benefits for a constituency” (McConnell, 2010), as well as Compton and ‘t Hart’s (2019) criterion of “costs/benefits associated with the policy are distributed equitably in society” should be reconsidered in relation to justice, because it may be unjust to create benefits for a constituency in a way that unduly burdens other societies, as is the case for colonial policies, for example.

Two tenets of justice apply to just program success, namely *distributive justice* and *recognition justice*. Distributive justice applies to program success, as the tenet pertains to the distributive effects of policies. As such, different principles of distributive justice can be leveraged to evaluate the policy goals and outcomes. In other words, to determine whether the programs were just, inspiration can be drawn from theories of justice, in which different principles of distributive justice, including non-Western principles depending on the context, are proposed as ‘rules’ or ‘yardsticks’ against which just program success can be assessed. As such, when evaluating policy programs through a distributive justice lens, the question becomes: are the outcomes distributed according to a contextually appropriate principle of distributive justice?

We can also evaluate the policy objectives, instruments, and outcomes through a recognition justice lens. This way, we can study what normative ideas and relations of recognition are implicitly reproduced or created through the policy programs. For example, Scheider and Ingram (1993) argued that policies ‘construct’ target populations, which may include stereotypes and the devaluation of certain social groups, which may harm people’s self-image or fail to respect participatory parity in social life. (Unconscious) Ideas about specific social groups being worth less or less deserving may result in policy objectives or instruments that can adversely impact these groups (House, 2017). As such, the injustice at stake is not merely maldistribution but also misrecognition.

In sum, both recognition justice principles can also be leveraged to evaluate policy programs. Given the three modes of (mis)recognition (i.e., love, status order, and dignity), policy programs can be evaluated from a recognition justice lens by asking the following three questions: (i) does the program facilitate or hinder bodily integrity and the development of healthy relationships with friends, family, and partners?; (ii) does the program devalue or ignore specific identities?; and (iii) does the program respect the intrinsic dignity, value, and rights of humans and non-humans?

Consequently, we propose the following definition of just program success:

*Just program success:* the policy program and its distributive outcomes are both successful and just, in which ‘justice’ is determined against a principle of distributive and/or recognition justice that can be reasonably defended in the specific context.

### Justice-incorporating notion of process success

Process success pertains to the process of policymaking and implementation. Some of the established criteria for process success include building a coalition in support of the policy, symbolizing innovation and influence, implementation in line with objectives, and little or no opposition to the process (McConnell, 2010). In a justice-incorporating notion of process success, however, the *decision-making processes are also just*. Again, against the backdrop of justice, existing justice-related policy success criteria may need amendments. For example, the criterion “stakeholders overwhelmingly experience the making and/or the delivery of policy as just and fair” (Compton &’t Hart, 2019) does not seem to be to be a necessary or a sufficient condition for justice, because stakeholders may evaluate just policies as unjust or altogether fail to experience injustices (van Uffelen & ten Caat, 2025). Moreover, the criterion of “robust deliberation and thoughtful consideration of the relevant values and interests” (Compton &’t Hart, 2019) may not apply to all political regimes, ideologies, and decision spheres.

In the context of just process success, both procedural and recognition justice apply. However, given the large variety of principles of procedural justice, there is normative uncertainty about whether specific decision-making processes are just or not. What is considered a just policy process in one venue, policy domain, or jurisdiction may not be considered just in another. For example, public participation in decision-making on energy production may be considered as just in the context of local renewable energy production, yet unjust in the context of nuclear energy (Winner, 2017). Moreover, the methods of participant selection, modes of communication, and degrees of authority granted to the public (Fung, 2006) may vary depending on the policy's nature but also on the stage of the policy process (i.e., agenda setting, policy formulation, policy adoption, implementation, and evaluation). For example, a policy that is considered just in agenda setting or issue framing may still be unjust in its implementation. Therefore, the question for evaluating policies in terms of justice becomes: Is the policy process just according to a contextually appropriate principle of procedural justice?

Moreover, recognition justice is relevant to evaluating decision-making processes, as situations justified by a principle of procedural justice might still increase misrecognition. For example, participatory processes might misrecognize citizens by portraying them as self-interested or epistemically unreliable (Rodhouse et al., 2021). Thus, misrecognition can occur even when the process is just according to an appropriate (democratic) principle of procedural justice. In sum, in policy processes, actors may be denied moral agency (misrecognition through dignity) or their input not valued or ignored (epistemic injustice, which can be considered a type of misrecognition through status order) (Fricker, 2007). As such, the following questions can help shape justice evaluations by incorporating recognition through status order and recognition through dignity: does the policy process assign appropriate credibility to the epistemic input of different social groups or devalue the input of some people due to biases or stereotypes?; and, does the policy process respect actors as decision-making agents capable of making moral decisions?

Therefore, we propose the following definition of just process success:

*Just process success*: the policy process is both successful and just, in which ‘justice’ is determined against a principle of procedural and/or recognition justice that can be reasonably defended in the specific context.

### Justice-incorporating notion of political success

The political dimension of the policy success heuristic acknowledges that policies also have political outcomes. Political success pertains to the political outcomes and impacts of a policy and occurs when one’s electoral prospects or reputation are enhanced; when one controls the policy agenda; when the broad values and direction of government are sustained; when opposition to political benefits is absent (Compton &’t Hart, 2019; McConnell, 2010). However, this view of policy success focused only on governments, even though—as highlighted by policy feedback theory (Mettler & Sorelle, 2018)—the political outcomes of policies affect all actors in society.

The categories of distributive justice and recognition justice apply to the political dimension. First, ‘just political success’ includes a reflection on the distribution of power. When the political outcomes result in too much power for certain political actors, there will be domination, or structural powerlessness (Young, 1990).^4^ This could be interpreted as a question of distributive justice (Walzer, 1983), and thus distributive justice principles can be leveraged to evaluate the (re-)distribution of power that follows from policies. The justice-evaluation question then becomes: do political outcomes lead to a just distribution of power according to a contextually appropriate principle of distributive justice?

Second, principles of recognition justice can also be leveraged to normatively evaluate the political outcomes of policies. On the one hand, policies can affect the standing of groups in society, for example, by changing the meaning of citizenship and altering membership in the community (Mettler & Sorelle, 2018). As such, political outcomes can be considered unjust through the mode of status order. On the other hand, when political outcomes result in a situation of powerlessness for some actors, this could constitute misrecognition through dignity. Here, powerless social groups can be susceptible to disrespect as moral agents capable of political decision-making, which constitutes harm to their relation-to-self (van Uffelen & Santos Ayllón, 2025). The questions for evaluating political success from a recognition lens (specifically, the modes of status order and dignity) become: do the political outcomes diminish the status of specific identities?; and, do the political outcomes respect actors as moral agents, capable of political decision-making?

As such, we propose the following definition of just political success:

*Just political success*: the political outcomes of the policy are both successful and just, in which ‘justice’ is determined against a principle of distributive and/or recognition justice that can be reasonably defended in the specific context.

### A framework for just policy success

Having outlined how justice intersects with programmatic, process, and political dimensions of policy success, we now turn to the question of how such evaluations can be operationalized. Evaluating policy outcomes, processes, and programs in terms of justice is complex, not least because of normative uncertainty, which implies that there are “situations where there are different partially morally defensible–but incompatible–options or courses of action, or ones in which there is no fully morally defensible option” (Taebi et al., 2020). As a result, normative choices need to be made concerning key aspects of the evaluation.

A central source of normative uncertainty is for whom justice is being evaluated (Marsh & McConnell, 2010; McConnell et al., 2020). To address this, it is essential to identify, with granularity, who the affected populations are. Here, intersectionality theory offers valuable guidance (Rainard et al., 2025). Rooted in critical social theory, intersectionality explains how inequalities are enabled and created by systems of discrimination and disadvantage (Crenshaw, 1991). It emphasizes that marginalization occurs at the intersections of social identities such as race, immigration status, and gender, and that policies designed by privileged groups often overlook the barriers that marginalized communities face. An intersectional analysis for policy evaluation consists of identifying—qualitatively or quantitatively—diverse identities and lived experiences to understand how systems of discrimination expose them to burdens of policy, and thus, co-constituting injustices (Rainard et al., 2025). In doing so, an intersectional lens helps generate a more comprehensive picture of social realities, which is crucial for evaluating policies through a justice lens.

As highlighted in the “On justice in relation to policy” section, another key source of normative uncertainty concerns the principles of justice that are considered relevant in each context (Van Uffelen et al., 2024). Distributive justice can mean many things in many different contexts, and people often disagree on how to distribute goods in society. Similar disagreements exist regarding procedural justice. For example, how much power should citizens have in decisions about local energy infrastructures? Or, are policy processes of the European Union democratically legitimate and—if not—how should they be reformed? More generally, different countries manifest different versions of democracy, aggregating votes, and procedures to amend constitutions, all of which reflect different conceptions of fairness. These variations illustrate that procedural justice is not self-evident, but subject to contestation.

**Fig. 1:**
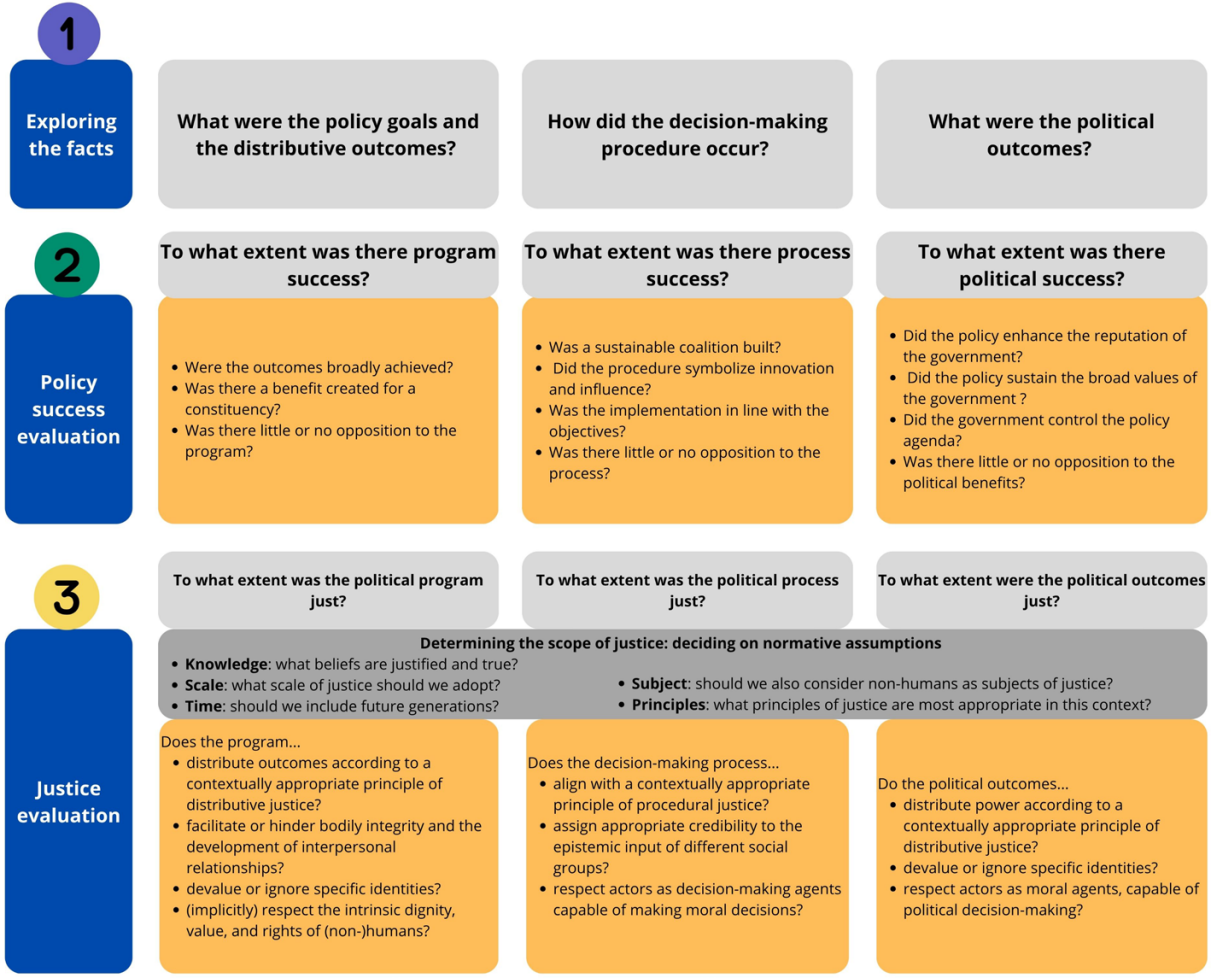
A holistic framework for policy evaluation, incorporating reflection questions for both policy success and policy justice

Given these complexities, we propose a reflective tool that synthesises questions posed by the policy success heuristic with those we articulated above “Justice-incorporating notion of program success” for navigating the key dimensions of justice-oriented policy evaluation (see Fig. 1).

The evaluation would start by articulating the boundaries of the evaluation. These include (Marsh & McConnell, 2010; Van Uffelen et al., 2024): (a) the scale of the evaluation, e.g., local, regional, national, or global; (b) the timeframe, e.g., how many future generations should we take into account; (c) knowledge, as in, the beliefs that are held to be justified and true; (d) actors, e.g., do our duties of evaluation extend to non-humans and ecosystems; and (e) the principles of justice, e.g., the criteria for determining what is (un)just. How these are identified and delineated is non-trivial, and what should be excluded or included is highly context specific.

Next, the evaluator should explore the ‘facts’ of the case, such as the objectives, instruments, and programmatic outcomes of the policy, the policymaking and implementation process, and the politics ensuing from the policy. Thereafter, relevant questions of the policy success heuristic can be selected and answered to arrive at an assessment of the degree of programmatic, process, and political success. Subsequently, the evaluation should address the questions posed above for each dimension of justice-oriented evaluation, i.e., programmatic justice, process justice, and political justice. Where a binary assessment of justice for any criterion is not sufficiently nuanced—similar to policy success—the evaluation could consider a spectrum from unjust to just. This should then be aggregated to an overall assessment of justice along each dimension and the policy more holistically.

## Discussion and conclusion

This study aimed to explore and further develop the normative aspects of policy evaluation. Building on the policy success heuristic and applied literature on justice, we constructed a reflective framework for evaluating policies in terms of just policy success. In doing this, we distinguish criteria associated with policy success from those that are associated with policy justice. Thus, we acknowledge that just policies may be unsuccessful and that successful policies may be unjust. Consequently, we offer a starting point for practitioners and researchers to evaluate policies in a more nuanced manner, with attention to both objective and normative aspects of policy evaluation.

Our framework makes three contributions to the literature. First, we add nuance to the ongoing discussion about justice in policy evaluation. While the policy success heuristic was a significant step toward bridging positive and normative approaches to policy evaluation, subsequent research has critiqued and enriched the heuristic by incorporating the notions of equity and procedural fairness within it. However, we show that these extensions do not fully capture the broader complexity of justice. For instance, programmatic success is often linked to distributive justice, but the latter cannot be reduced to a notion of equity. Similarly, procedural justice cannot be reduced to participation or inclusion, particularly across diverse cultural and political contexts. Moreover, the absence of opposition to a policy does not imply that it is just, as a lack of resistance may reflect political marginalisation or limited access to information. Our framework highlights that both distributive and procedural justice are multifaceted and should be evaluated using principles that are contextually appropriate.

Second, we highlight the relevance of recognition justice as a critical lens in policy evaluation. As Schneider and Ingram (1993) have argued, policies not only distribute resources but also ‘construct’ affected populations; they might intentionally or unintentionally misrecognize some groups in society, for example, through stereotyping or stigmatization. Thus, whether policy processes, programs, and outcomes perpetuate injustices from a recognition perspective is an important question for policy evaluation. Recognition justice broadens the normative scope of evaluation beyond distribution and procedure, drawing attention to how policies influence human dignity and (de)value social identities.

Third, our framework brings a justice lens to the evaluation of political success, an area that has remained undertheorized. While McConnell et al. (2020) have acknowledged that political success depends on the perspectives of various stakeholders, these insights have not been systematically connected to normative theories of justice. Drawing also on policy feedback theory, we propose that political outcomes should be evaluated in terms of how they redistribute political power, reshape the standing of groups in society, and alter civic agency. We show that both distributive and recognition justice offer valuable entry points for understanding whether political outcomes contribute to or undermine justice.

From our framework, it follows that there may be interdependencies—whether synergies or trade-offs—between policy justice and policy success. Even as successful policies can contribute to injustice, just policies can fail on conventional success criteria. For example, while a subsidy program for solar energy may increase renewable energy adoption (programmatic success), it may be considered unjust if only high-income citizens can benefit from the policy. Conversely, a redistributive income tax may reduce inequities in society but result in political failure. In a more complex scenario, it is possible that—while meeting its stated policy objectives (programmatic success)—a policy program worsens distributive inequities (distributive injustice) even though its objectives reflected the preferences of marginalized communities (procedural justice). Our framework facilitates a critical reflection on the synergies and trade-offs between justice and success in such cases, rather than assuming alignment between the two.

The relation between different categories of justice is complex. While we propose treating distributive, procedural, and recognition justice as analytically distinct, we acknowledge that injustice in one dimension can reinforce or trigger injustice in another dimension. For instance, the failure to consider vulnerable groups during agenda setting (procedural injustice) may result in a policy program that devalues their identities (recognition injustice) and worsens their economic position in society (distributive injustice). At the same time, inequitable socioeconomic distribution or structural misrecognition may limit political voice, creating procedural injustice. These dynamics suggest that evaluation should attend to each justice dimension separately, while also examining how they interact empirically.

While we focused on developing criteria for evaluating justice in the context of public policy, it is equally important to consider the processes through which such evaluations are conducted. The operationalization of justice—such as selecting appropriate principles, determining relevant metrics, or deciding the timeframe—inevitably involves normative choices. As mentioned earlier, scholars have long emphasized that evaluation itself is a political and ethical act. To make this explicit, we framed our approach as a set of reflective questions rather than a fixed template of principles, metrics, or yardsticks. These decisions may be made by analysts, evaluators, policymakers, or other stakeholders, either independently or in collaboration. House and Howe (1999), for instance, have argued that a just evaluation must be grounded in deliberative democratic principles, characterized by inclusive dialogue and critical engagement among diverse social groups. However, as expounded in the “Recognition justice” section, in some contexts other principles may also be defensible for guiding a just policy evaluation process.

We encourage analysts, evaluators, policymakers, and researchers to adapt and use the reflective framework not only as a tool for assessing justice in public policy, but also for furthering the broader discussion on what constitutes a just approach to evaluation itself. In doing so, we hope to contribute to ongoing dialogue about how to evaluate justice—and how to evaluate justly.

## Data Availability

No datasets were generated or analysed during the current study.

## Footnotes

1 The attribution to institutions distinguishes the concept of justice from the more overarching concept of ‘goodness’, which applies to both institutions and actions of individuals.

2 We avoid the word ‘subjective’ here, because that seems to imply that justice is like ‘flavours’ and every claim of injustice is equally justified, which leads to a relativism that is unproductive. Instead, we consider justice as plural and contested, as there are multiple conceptions of justice that can be (in)appropriate in specific contexts, and there can be stronger or weaker reasons to consider things as (un)just in specific contexts.

3 Unjust values can be institutionalised intentionally, or unintentionally, for example, if policy makers lack understanding about the affected populations.

4 The notion of ‘too much’ is vague on purpose, as there may be normative uncertainty about how much is ‘too’ much’ in different political regimes.
